# Population age and household structures shape transmission dynamics of emerging infectious diseases: a longitudinal microsimulation approach

**DOI:** 10.1098/rsif.2023.0087

**Published:** 2023-12-06

**Authors:** Signe Møgelmose, Laurens Vijnck, Frank Neven, Karel Neels, Philippe Beutels, Niel Hens

**Affiliations:** ^1^ Data Science Institute, Interuniversity Institute of Biostatistics and statistical Bioinformatics, Hasselt University, Hasselt, Belgium; ^2^ Center for Population, Family and Health, University of Antwerp, Antwerp, Belgium; ^3^ Centre for Health Economic Research and Modelling Infectious Diseases, Vaccine & Infectious Disease Institute, University of Antwerp, Antwerp, Belgium; ^4^ School of Public Health and Community Medicine, The University of New South Wales, Sydney, Australia

**Keywords:** mathematical modelling, demography, population ageing, epidemiology, disease transmission, household structures

## Abstract

Host population demographics and patterns of host-to-host interactions are important drivers of heterogeneity in infectious disease transmission. To improve our understanding of how population structures and changes therein influence disease transmission dynamics at the individual and population level, we model a dynamic age- and household-structured population using longitudinal microdata drawn from Belgian census and population registers. At different points in time, we simulate the spread of a close-contact infectious disease and vary the age profiles of infectiousness and susceptibility to reflect specific infections (e.g. influenza and SARS-CoV-2) using a two-level mixing model, which distinguishes between exposure to infection in the household and exposure in the community. We find that the strong relationship between age and household structures, in combination with social mixing patterns and epidemiological parameters, shape the spread of an emerging infection. Disease transmission in the adult population in particular is to a large degree explained by differential household compositions and not just household size. Moreover, we highlight how demographic processes alter population structures in an ageing population and how these in turn affect disease transmission dynamics across population groups.

## 1. Background

Host population demographics and patterns of host-to-host interactions are important drivers of heterogeneity in infectious disease transmission. To describe the dynamics of infections transmitted via close-contact interactions, particular attention has been given to social mixing patterns, which can be captured by demographic structures. The frequency and pattern of social mixing with relevance for the spread of close-contact infectious diseases are highly dependent on age. Children, teenagers and young adults have more contacts and are disproportionately more likely to mix with people of their own age than with adults older than 25 years. Adults also display age-assortative mixing, but their average contact frequency is lower and their contacts are less concentrated in their own age group than those of youngsters [1,2]. Consequently, age patterns are seen in susceptibility and exposure to many pathogens [3]. Additionally, changes in the immune system throughout the life course can add to these age-specific differences. Children tend to be more susceptible to infections given that their immune system is still maturing, while in older adults, the ageing of the immune system (immunosenescence) may increase their susceptibility to infection and to more severe disease upon infection [4]. Likewise, the infectiousness of infected individuals may also vary by age [5,6].

Population structures beyond age further add to the heterogeneity in social mixing patterns and in disease transmission dynamics. Due to the high frequency, long duration and intimacy of within-household contacts, household transmission constitutes a substantial risk factor in infectious disease dynamics [7,8]. Moreover, households often contain people from different generations (e.g. parents and children) belonging to different subpopulations outside the household, which, for example, can facilitate the spread of an infection from schools to workplaces [9]. Consequently, age- and household-structured models of infectious disease transmission with social mixing have proven highly valuable for modelling the transmission of close-contact infectious diseases (e.g. [10–15]).

Still, it remains challenging to model an age- and household-structured population, and in particular the changes therein, in a well-founded and feasible manner. Detailed household data are usually unavailable, which often makes it necessary to recreate households by relying on probability matching and/or to make simplifying assumptions, like limiting to specific household sizes or types (e.g. nuclear families). Less common living arrangements such as long-term care facilities (LTCFs) or multi-generational households are often disregarded, although they may be important for disease transmission [16]. Moreover, only few studies incorporate evolving age and household structures in the host population or consider multiple populations with different compositions (e.g. [11,17–20]). While demographic change can be reasonable to disregard when the period under consideration is short, it may be necessary to allow for changing population structures when investigating disease transmission dynamics and control strategies in different populations or over a longer time frame (i.e. years or decades depending on the population, infection and research question), where demographic changes become more apparent. Moreover, a thorough understanding of the relationship between disease transmission dynamics and host population structures, as well as the demographic processes underlying these structures, may be important for assessing how future demographic changes potentially could affect transmission dynamics.

Demographic change has in many countries led to an increasing median age of the population (population ageing), which has become a global phenomenon [21]. Many of the most developed countries have been ageing for decades as a result of declining fertility rates and increasing life expectancy, and the ageing of the large generations born in the mid-twentieth century is currently causing a temporary acceleration of population ageing [22].

We investigate how emerging infections are spreading in a relatively old (i.e. high median age), and still ageing, host population. We use longitudinal microdata drawn from Belgian census and population registers, including individual-level information on age, sex, household membership and kinship links. The data feed into a demographic microsimulation, which includes dynamic demographic processes for fertility, mortality, migration and household transitions, making it possible to model the Belgian population over time with evolving age and household structures.

We subsequently combine the demographic microsimulation with a two-level mixing model, which distinguishes between exposure to infection in the household and exposure in the community at large. We base contact networks within households on empirical data, rather than making the common assumption of random mixing. We simulate the spread of an emerging close-contact infectious disease in 2020, 2030, 2040 and 2050, which allows sufficient time for noticeable changes in age and household structures to emerge. Furthermore, we vary the age profiles of infectiousness and susceptibility to reflect specific infections, including influenza and SARS-CoV-2.

We aim to explore how the relationship between age and household structures affects disease transmission dynamics of an epidemic at the individual and the population level. Moreover, we show how demographic processes alter the population structures in an ageing population and investigate how this affects the transmission dynamics across population groups.

The paper is organized as follows: in §2, we give specific details on the demographic microsimulation, including the demographic data and processes considered. Similarly, we describe the disease transmission model with two levels of mixing along with the model parameters. Section 3 presents the population structures and changes therein and documents the disease incidence by age and household composition. Furthermore, the impact of epidemiological heterogeneities within the population is visualized in a scenario analysis. Finally, in §4, we discuss the results as well as the strengths and limitations of the study.

## 2. Methods

We model the host population and the spread of an infection at the individual level using microsimulation, also referred to as individual-based modelling or agent-based modelling [23]. Each individual in the population is represented explicitly and assigned a set of relevant attributes (e.g. age, sex, marital status, household membership, disease state). The population evolves over time as a result of individual events and events emerging from interactions between individuals (e.g. marriage, death, social contact, disease transmission). All events are tied to the individual, meaning that the life course and health trajectory of each person is tracked [24]. Consequently, outbreaks at the population level emerge from the interactions between the individuals. Next, we describe the initial population and the processes used to determine the occurrence of demographic events and disease transmission events.

### 2.1. Demographic microsimulation

We developed a demographic microsimulation to simulate the Belgian population from 2011 to 2051. The initial population in the microsimulation is based on the Belgian census from 1 January 2011, from which we drew a household-based sample corresponding to about 10% of the total population. For each individual, we have information on their date of birth, sex, coded ID of parents, birth trajectory (parity and date of most recent birth if applicable), household ID and household position (e.g. in union, child, single parent). Thus, individuals can be linked to each other through household membership and kinship.

In each time step (i.e. day), individuals can enter and leave the population as a result of births, deaths and migration. Moreover, the household position of an individual may change and transitions between households or the creation of a new household can take place. Household transitions include children leaving the parental household, union formation or dissolution and older adults moving to LTCFs. The household transition and other demographic events of one individual may thus also affect the household position of other individuals. A single parent, for example, changes household position to single after the last child moves out and an individual in a union becomes single (parent) after their partner dies.

The demographic events take place by comparing an individual's probability of a given event with a random number between zero and one. The event is executed if the probability is larger than or equal to the random number. The probability of a demographic event taking place varies by individual characteristics, including age, sex and household position, and changes over time except for the household transition rates. Finally, ageing takes place at the end of the time step and the population is updated accordingly.

We assume that mortality, fertility and migration levels in the microsimulation resemble the observed and projected rates by the Belgian Statistical Office (StatBel) and the Belgian Federal Planning Bureau (FPB). This implies below-replacement level fertility (i.e. a total fertility rate (TFR) below 2.1 [25]), as the TFR in the simulation is decreasing from approximately 1.8 in 2011 to 1.5 in 2020 followed by a slow increase to about 1.62 by 2050. Moreover, we assume continuous improvements in longevity, especially for males for which the life expectancy at birth is increasing from 77.7 years in 2011 to 85.4 years in 2050 [26,27]. Consequently, the population will continue ageing, with implications for the household structures.

We keep track of all demographic events in an event log file, which makes it possible to recreate the population and the changes therein. The host population in the disease transmission model thus evolves in a deterministic manner, making it possible to solely ascribe differential outcomes in a given simulation year to the disease transmission parameters.

The demographic data, model and source code are described in detail in the electronic supplementary material.

### 2.2. Disease transmission model

Although we allow demographic events to take place on a daily basis, substantial effects of population ageing will only emerge after several years, as demographic change is a slow process. We have therefore chosen to simulate disease outbreaks every 10 years. In 2020, 2030, 2040 and 2050, 10 randomly chosen individuals become infected, in an otherwise fully susceptible population, on 1 January of each respective year. We consider an infectious disease transmitted via close contact, which can be represented by a susceptible–infectious–recovered (SIR) model, and consider several scenarios for age-specific susceptibility and infectiousness. The probability of becoming infected, and thus moving from the susceptible to infectious state, is calculated using a two-level mixing model, where an individual can acquire infection as a result of interactions with an infected household member (local contact) or an infected individual in the general population (global contact) [28]. For each combination of parameter settings in the two-level mixing model, we run 30 simulations, but in the analysis, we disregard those where an outbreak never takes place (i.e. total attack rate of less than 0.5%).

#### 2.2.1. Within- and between-household interactions

We use a contact matrix to estimate social interactions (i.e. a proxy for an at-risk event at which infection can be transmitted) between non-household members in the general population. The contact matrix is based on social contact data collected in a survey in Belgium in 2010–2011 [2] and made available through the SOCRATES data tool [29] (electronic supplementary material, figure S1). Contacts between household members were excluded, as these are captured by the household level of the model, but contacts taking place in the household with non-household members were included. Additionally, supplementary professional contacts (SPC) were excluded.

To model interactions among household members, we construct a household contact network for each household in the population. Specifically, each household member is represented by a vertex, and a link between two vertices indicates a contact between those two household members. The links are constructed using an exponential random graph model developed by Krivitsky *et al*. [30], which was fitted to data from two contact surveys conducted in Belgium in 2010–2011 [2,9]. The model accounts, among other things, for the type of household and the age-sex composition. The probability of a contact between two household members, however, is independent of past contacts between them. In each time step (i.e. day), we apply the fitted model from Krivitsky *et al*. [30] to the households in the simulated population and simulate who comes into contact with whom within each household. The mean network density (the number of links in a household relative to the number of possible links [31]) by household size and type are shown in electronic supplementary material, figure S2. Contacts between household members are often repetitive because of the high contact density in the households. In the general population, repetitive contacts are less likely because of the large population size. Nevertheless, they may still be important, but data on the share of repetitive contacts are lacking.

#### 2.2.2. Risk of infection

Each susceptible individual *i* acquires infection at time *t* with probability *p_i_*(*t*),

2.1 $$\begin{array}{c} \;p_{i}(t)=1-\underset{\;j\neq i\;j\in h_{i}}{\prod }(1-\beta_{h}s_{i}z_{j}a_{ij}(t)I_{j}(t))\cdot \underset{\;j\notin h_{i}}{\prod }(1-\beta_{p}s_{i}z_{j}c_{ij}(t)I_{j}(t)), \end{array}$$

where *h_i_* denotes the household of individual *i* and the parameters *β_h_* and *β_p_* represent the probabilities of disease transmission given contact between a susceptible and infectious individual within the household and in the general population, respectively. We vary the transmission parameters to reflect different settings (e.g. high versus low household transmission). The relative susceptibility and infectiousness given the ages of individual *i* and *j* are represented by *s_i_* and *z_j_*, respectively, while *I_j_*(*t*) takes the value one if individual *j* is infected at time *t* and zero otherwise. The contact network in household *h_i_* is represented by an adjacency matrix *A* of which the element *a_ij_*(*t*) equals one if household members *i* and *j* come into contact with each other at time *t*, and zero otherwise. A new adjacency matrix is generated in each time step.

The social contact matrix from electronic supplementary material, figure S1 contains the mean number of contacts per day in the general population between each age group, *m_ij_*, thus we compute the probability by which individual *i* and *j* come into contact with each other on a given day (time *t* is discretized in days) based on the age groups to which *i* and *j* belong, *c_ij_*(*t*), as follows:

2.2 $$\begin{array}{c} c_{ij}(t)=\frac{m_{ij}}{N_{j}(t)}. \end{array}$$

We assume disease transmission in the general population to be frequency-dependent, meaning that the average number of effective contacts made by each person remains unchanged as the population grows. Thus, to keep the age-specific contacts constant over time, the element *m_ij_* is divided by *N_j_*(*t*), the size of the age group of *j* at time *t*. In each time step, the probability of infection (1) is computed for all susceptible individuals in the population and their disease state is updated accordingly.

We are not taking factors like seasonality, weekends and school holidays into account, as we are focusing on the role of population heterogeneity in the spread of an infection. The interplay between these factors remain a topic for further research.

#### 2.2.3. Infectious period

We assume that the infectious period follows a gamma distribution with a mean of 3.8 days and a standard deviation of 2 days, reflecting the infectious period for influenza [7,32,33]. For each newly infected individual, a value is drawn from the distribution and rounded to the nearest integer. An infected individual recovers and obtains immunity when the infectious period has passed.

#### 2.2.4. Age-specific susceptibility and infectiousness

**Figure 1.**
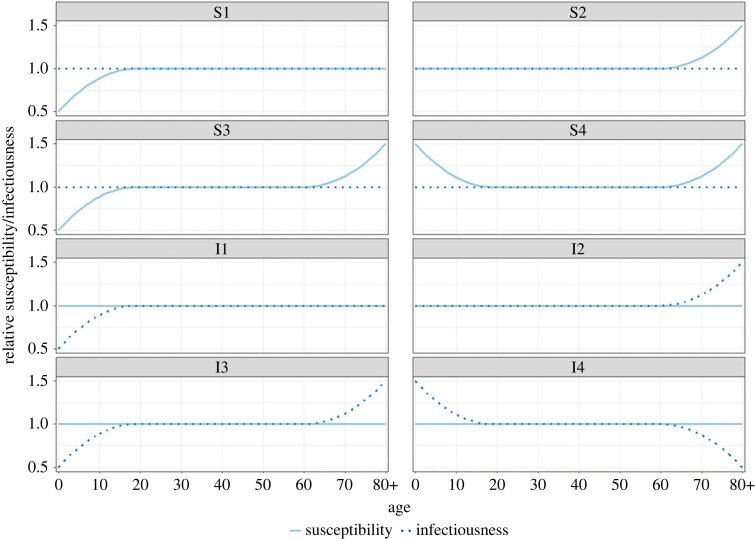
Age-specific susceptibility and infectiousness scenarios.

We consider different scenarios for age-specific susceptibility and infectiousness. Whereas this is primarily an explorative set of scenarios, some of these age-specific susceptibility and infectiousness profiles were motivated by specific infections, including influenza and respiratory syncytial virus (RSV) (e.g. scenario S4 [34]), SARS-CoV-2 (e.g. scenario S1 or S3 [35–37]). Other considered elements include the general phenomenon of immunosenescence depicting higher susceptibility at older age (e.g. scenario S2). Moreover, we use the scenarios to assess the role different population groups play in the spread of an infection. The age-specific susceptibility and infectiousness scenarios are shown in figure 1 in relative terms, meaning that a value below one corresponds to reduced susceptibility or infectiousness and a value larger than one implies increased susceptibility or infectiousness for individuals in the given age group. Susceptibility is age-dependent (different from one) in scenario S1–S4, while infectiousness is age-dependent in scenario I1–I4. We compare the different scenarios with a baseline case where infectiousness and susceptibility are equal across all ages.

## 3. Results

### 3.1. Population dynamics

**Figure 2.**
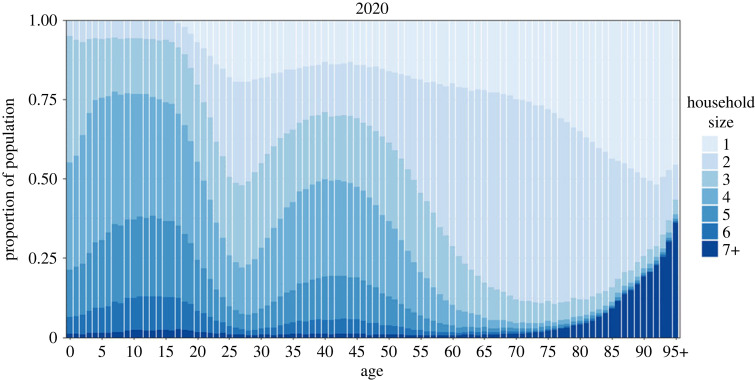
Household size distribution by age group of simulated population in 2020.

Age and household size are closely connected as seen in figure 2. Children and adolescents most often live with their parent(s) and siblings, meaning that households of size three and larger are most common at young ages, which implies a similar pattern in the parental age groups (e.g. ages 30–55). The average household size starts to decrease in late adolescence, as children leave the parental household, and increases again from the late twenties with the entry into parenthood. Again, a similar pattern is seen in the parental generation (e.g. age 50+), but with a continuous decrease in mean household size until the mid-seventies, when widowhood and the need for LTCFs become more prevalent. Consequently, single-person households and very large households (i.e. LTCFs) become more frequent in the oldest age groups.

**Figure 3.**
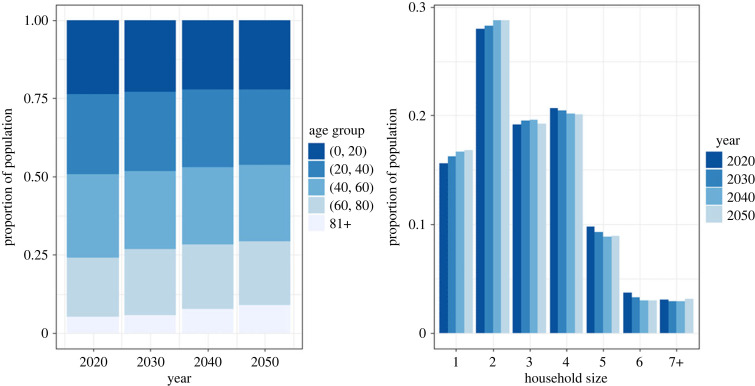
Age distribution (left) and household size distribution (right) of the simulated population.

The population structures do not change dramatically over time, as the Belgian population in 2020 already has an old age structure and we are not assuming extreme changes in the vital rates in the simulation period of 30 years. Nevertheless, the simulated population continues ageing between 2020 and 2050 as seen in figure 3 (left panel). The share of the population older than 60 years increases by 22% between 2020 and 2050, while the share in the oldest age group alone (i.e. 81+) increases by more than 75%. This is the result of past long-term trends of declining fertility and increasing longevity, which continue to a certain degree in the simulation period. Moreover, the ageing of the large generations born in the mid-twentieth century causes a temporary acceleration of the population ageing. The changes in the age distribution are also reflected in the household size distribution as seen in figure 3 (right panel). The proportion of the population living in a single-person household increases as the population ages, since the proportion of people living alone is higher in the older age groups. The proportion living in households of size two is increasing from 2020 to 2040, which is mainly due to the increased survival of elderly males in a union.

While an increasing share of the population lives in small households of size one to three, the proportion of larger households of size four to six is decreasing. Households of those sizes are to a large extent occupied by nuclear families in the age range of 0–50 years (see electronic supplementary material, figure S3–S4), which makes up a decreasing proportion of the simulated population from 2020 to 2050. The proportion of the population living in households of size seven and larger remains quite stable during the simulation period, but it is the result of two opposite trends. The proportion of parents with a large number of children, and therefore with a large household, is decreasing, while people living in LTCFs (i.e. elderly population), make up an increasing proportion of the population.

The age-specific household size distributions slightly change over time (see electronic supplementary material, figure S3–S4). The average household size for children and their parents (i.e. younger than 50 years) is decreasing. This is a consequence of single parent families becoming more prevalent and a decreasing TFR prior to 2020, followed by a slow, but not full, recovery (see electronic supplementary material, figure S5). Meanwhile, the average household size in age group 50–70 is increasing as the likelihood that their household accommodates (adult) children increases, due to past changes in the timing of childbearing. A change in the household size distribution is also seen in the elderly population as a result of improvements in longevity. Consequently, single-person households and collective households (i.e. LTCFs) are increasingly being replaced by two-person households.

### 3.2. Disease transmission dynamics

#### 3.2.1. Attack rate

The proportion of the population that contracts the infection during an outbreak (the attack rate) responds to changes in the transmission parameters (*β*_h_ and *β*_p_ in equation (2.1)) in a nonlinear pattern (see electronic supplementary material, figure S6). The relative increase in the attack rate diminishes as the transmission probabilities increase, especially when the transmission probability given contact within the household increases. Potential household infections are limited by the size of the household and more than 15% of the simulated population live in single-person households. Thus, as the household transmission probability keeps increasing, everyone with an infected household member will eventually become infected as well.

The attack rates in each susceptibility and infectiousness scenario deviate from the baseline scenario since some population groups face an increased or decreased risk of acquiring or transmitting the infection given the specific scenario. However, the differences between the scenarios diminish as the transmission probabilities increase.

#### 3.2.2. Age- and household-specific transmission dynamics: baseline scenario

**Figure 4.**
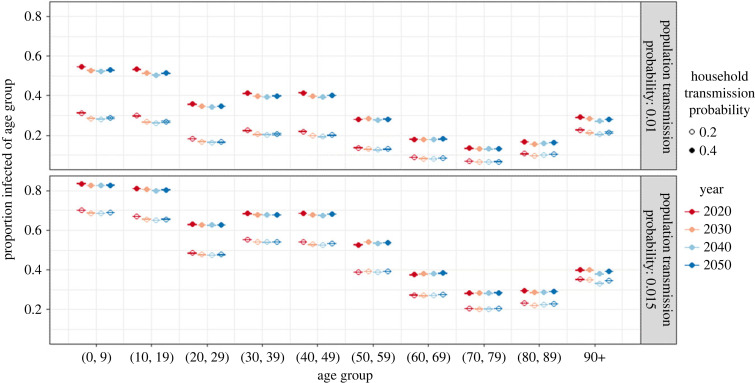
Mean and 95% bootstrap confidence interval for the mean age-specific attack rate for varying transmission parameters (*β_h_*: filled versus open circle, *β_p_*: upper versus lower panel) in the baseline scenario (susceptibility and infectiousness are equal across age).

The age-specific attack rates in the baseline scenario are shown in figure 4 for varying probabilities of household (closed versus open circle) and community transmission (upper versus lower panel) and at the different time points. We see that some population groups are more likely to get infected than others, also when discarding age-specific differences in susceptibility and infectiousness. The proportion of children and adolescents getting infected is larger than that of any other age group. The adult population also faces relatively large attack rates, which decrease from age 50 onwards. This reflects the age pattern in social contacts outside the household (see electronic supplementary material, figure S1).

Nevertheless, social mixing in the general population alone cannot explain the age distribution in the attack rates. The age group 20–29, for example, has more contacts in the general population than the age group 30–49, yet lower attack rates. This is due to the difference in household composition of these age groups. Individuals in their 20s are much more likely to live in households of size one or two, and thereby have a lower number of possible household contacts than people of age 40–49, who often live in larger households, as seen in figure 2.

Moreover, the household members of the two groups tend to differ in case of a larger household. People aged 30–49 living in a household of size three or larger often have young children living with them, while the 20- to 29-year-olds are more likely to live together with their parents or unrelated adults in a house-sharing arrangement (see electronic supplementary material, figure S4). The mean density of the contact network is higher in the first household composition than in the latter because of the presence of young children (see electronic supplementary material, figure S2). Finally, children and teenagers are more likely to bring an infection into the household given their high number of contacts in the general community, including schools, thus putting the parents at an increased risk (assuming that susceptibility and infectiousness are independent of age).

The attack rates in the oldest age groups can also only be explained by considering household structure. The attack rate is higher in age group 90+ than in 80–89, despite both age groups having identical social contact rates outside the household. From the age of 80 onwards, small households of size one and two are increasingly replaced by very large households (figure 2), such as LTCFs.

**Figure 5.**
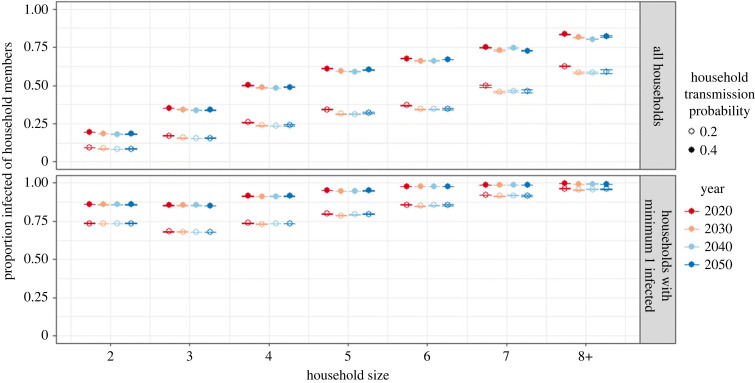
Mean and 95% bootstrap confidence interval for the mean proportion of household members getting infected by household size in baseline scenario with population transmission parameter of 0.01. Upper panel: estimate based on all households. Lower panel: estimate based on households with minimum one infected individual.

Household-specific attack rates (i.e. the proportion of household members acquiring infection) are shown in figure 5 by household size (x-axis) and household transmission probability (open versus closed circle). Moreover, we distinguish between a risk set containing all households (upper panel) and a risk set only containing households with at least one infected household member during the outbreak (lower panel). Generally, the household-specific attack rate increases by household size (figure 5 upper panel). This result is a combination of how likely an infection is to enter the household and how easily it spreads within that household. The number of individuals that can bring an infection into the household increases with the household size; however, the likelihood of it happening also depends on the social contact patterns of each household member. The infection is more likely to spread to households with at least one child younger than 13 years than to households of the same size without children, because children have a relatively large number of contacts in the community (see electronic supplementary material, figure S7).

After the infection has entered a household, the further spread is affected by the household size and composition. The within-household transmission is visualized in the lower panel of figure 5, as the estimates are limited to households with at least one infected individual. In that case, the differences across household sizes are substantially smaller and the mean proportion of household members getting infected even decreases from household size two to three. The decrease, however, is only observed for households without a young child. The presence of a child in the household affects the within-household transmission across all household sizes of less than eight, as young children tend to have more contacts with their household members (i.e. parents, siblings) than teenagers and young adults do with theirs (see electronic supplementary material, figure S8).

#### 3.2.3. Effect of demographic change on transmission dynamics: baseline scenario

The proportion of the population acquiring the infection during an outbreak is decreasing from 2020 to 2040 in all scenarios but the trend stabilizes or reverses between 2040 and 2050 (see electronic supplementary material, figure S6). This is the result of changing household structures and population ageing. The elderly population, which over time makes up an increasing proportion of the population, has relatively few contacts on average since the majority lives in small households and has few contacts in the general population. Consequently, the elderly population has a lower risk of acquiring and transmitting an infection than younger age groups.

Additionally, the changing household compositions in the population younger than 50 years of age resulting from low fertility levels and an increasing proportion of single parent families, decreases their risk of infection over time, with implications for the overall incidence. Meanwhile, the proportion infected of age group 50–70 remains more or less stable, despite an increasing proportion living in households larger than size two. Finally, improved longevity implies that the elderly population (especially females) becomes more likely to live with their partner than alone or in LTCFs, which affects the incidence in the oldest age group (90+).

The relationship between risk of infection and household size persists as the population is ageing, but the proportion of infected household members is decreasing over time across all household sizes (figure 5 upper panel). Meanwhile, the within-household transmission remains stable over time (figure 5 lower panel).

#### 3.2.4. Effect of age-specific infectiousness and susceptibility

**Figure 6.**
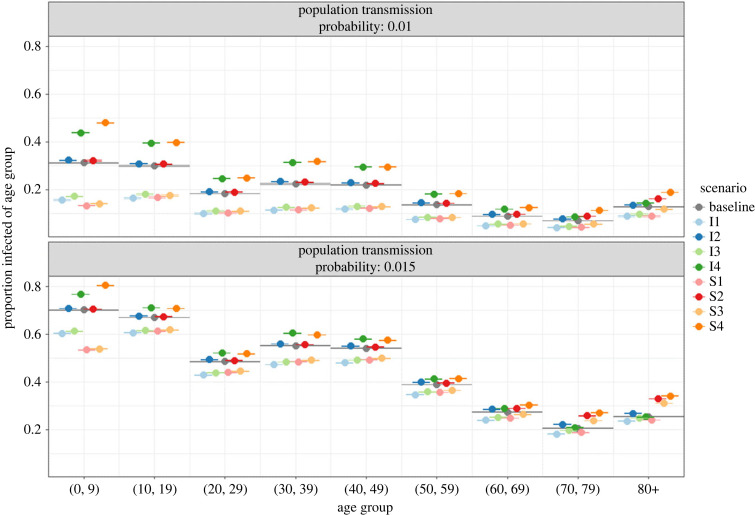
Mean and 95% bootstrap confidence interval for the mean age-specific attack rate in baseline scenario (grey) and each susceptibility/infectiousness scenario across varying population transmission probabilities (upper versus lower panel) and household transmission probability of 0.2. Simulation year 2020.

We further investigate the role different age groups play in the spread of an infectious disease. In figure 6, we compare the age-specific attack rates in 2020 across the susceptibility and infectiousness scenarios with those of the baseline scenario (i.e. corresponding to figure 4). This is visualized for varying population transmission probabilities (upper versus lower panel).

Differences from the baseline attack rate are not only seen in the age groups with modified susceptibility or infectiousness, but also in the rest of the population to varying degrees. The susceptibility and infectiousness of children affect all population groups, and the parental generation in particular (e.g. age group 30–39), to a markedly larger degree than changes in the susceptibility and infectiousness of the elderly population. In scenario S1 (I1), children have a relatively low susceptibility (infectiousness) which affects all other age groups substantially, while the relatively high susceptibility (infectiousness) from age 65 onwards in scenario S2 (I2) has a much smaller effect on the incidence in other age groups.

This is also seen by comparing scenarios S1 and S3 (I1 and I3), where the attack rates below the age of 60 do not differ substantially, despite the increased susceptibility (infectiousness) of the elderly population in the latter scenario. Even the elderly population itself is only somewhat affected by changes in their infectiousness. However, it applies to children as well as the elderly, that a change in their infectiousness only has a slightly larger impact on the incidence in the rest of the population compared with the same change in susceptibility.

As the population transmission probability (upper versus lower panel in figure 6) increases, the absolute difference between each scenario and the baseline attack rate diminishes, except for the age group 70 and older when subject to increased susceptibility, as in scenarios S2, S3 and S4. This is particularly pronounced in scenario S3, where children have relatively low susceptibility while the old age groups have high susceptibility. As the transmission probabilities increase, the attack rates in age groups 70–79 and 80+ in S3 shift from being below to being above those of the baseline scenario. The attack rates change slightly over time in all scenarios, but the position of each scenario relative to the other scenarios and the baseline remains unchanged (electronic supplementary material, figures S9 and S10).

## 4. Discussion

An understanding of demographic structures in the host population and how these influence disease transmission can be important for determining which population subgroups are most at risk and most effective to target in an intervention [38]. Moreover, an understanding of the demographic processes underlying the population structures may be important for assessing how future demographic changes potentially could affect transmission dynamics.

Using longitudinal microdata drawn from Belgian census and population registers, we model a host population with evolving age and household structures using microsimulation and illustrate how population ageing and changing household dynamics may further unfold in the next decades. We combine the demographic microsimulation with an epidemic model with two levels of mixing and illustrate a strong link between age and household structures and the implications thereof for the risk of infection for different population subgroups during an epidemic. Additionally, we quantify the potential impact of changing age and household structures on disease transmission.

The age structures in the social contact patterns are mirrored in the age-specific attack rates as the youngest age groups, with the most community contacts, have the highest risk of infection. The attack rates in the adult population, however, are to a larger degree explained by the differences in household compositions between young adults, middle-aged adults and older adults, which are related to the timing of demographic events. Young adults in their 20 s face a relatively low risk of infection on average, but it increases with the entry into parenthood, when assuming that susceptibility and infectiousness are independent of age. The child will eventually have a relatively high number of contacts outside the household (e.g. in day-care, school) as well as frequent contact with the parent(s) within the household, making the risk of infection high compared with other population groups. These relationships change to some degree in the different scenarios for age-specific infectiousness and susceptibility.

The incidence in families with children and/or adolescents decreased during the simulation period as a consequence of changing household compositions. In the decade prior to the first outbreak simulations, 2010–2019, the TFR decreased followed by a slow, but not full, recovery during the remaining simulation period, similarly to observed and projected rates by StatBel and FPB [27]. Changing fertility levels combined with an increase in single parent families affected the household compositions and indirectly disease transmission. This also affects disease transmission in other population groups since families with (school-age) children are important drivers in an epidemic.

As the children grow up and, in most cases, eventually leave the parental household, the number of household contacts of the now middle-aged parental generation is decreasing again, often in combination with decreasing community contacts, leading to lower attack rates. However, we found that the proportion of middle-aged people with (adult) children living in their household is increasing during the simulation. This change is related to the postponement of parenthood since the probability of leaving the parental household is assumed to be constant over time. Parents are increasingly older when the last child leaves the parental household because the average age at childbirth was increasing prior to 2011 when our simulation begins [39]. Several years later, these past fertility trends affect the household composition of the middle-aged population and indirectly their risk of infection. The increasing household size was expected to increase the risk of infection in the middle-aged population, but the effect is more or less counterbalanced by the decrease in the overall incidence induced by the changing household compositions in the younger age groups.

It should be noted that the relative distribution of births by the age of the mother is only slightly changing in the first decades of the simulation and eventually stabilizes. However, the average age at childbirth in Belgium has increased since 2011 and this is expected to continue in the future, to some degree [27]. Hence, the average age at childbirth, and indirectly the age at which children leave the parental household, is likely to increase more than in our microsimulation.

The risk of infection in the elderly population was also found to be highly dependent on their living arrangement. Community contacts are decreasing with age and a large proportion of old people live alone or only with their partner, which minimizes the number of occasions where transmission of a close-contact infection can take place. However, from the age of 80 onwards, the proportion of the population living in LTCFs, which tend to be very large households, increases considerably and so does the risk of infection. Consequently, the age pattern in the attack rates in the elderly population is shaped by the proportion living in collective households.

During the simulation period, collective households and single-person households in the population older than 80 years of age are increasingly being replaced by two-person households, as a result of improved longevity, particularly of males. We assume that the sex differential in mortality decreases during the simulation period as a result of larger improvements in men's mortality than in that of women. This implies that an increasing proportion of elderly women are living with a partner instead of alone, which intuitively should increase their risk of infection. However, the probability of moving to LTCFs, which are very large households associated with a high risk of infection, is substantially higher for elderly people living alone than for those living with a partner. Consequently, gradually fewer elderly people move to LTCFs and therefore incur a substantially lower risk of infection.

The future living arrangements and mortality of the elderly population are, like all other demographic processes, associated with uncertainty [40]. However, the sex differential in mortality has been decreasing and this is considered likely to continue, to some extent, in the future. The resulting changes in the household structures of the elderly population seen in our microsimulation are in agreement with existing studies of past and future trends in the living arrangements and mortality of older adults [41,42].

In addition to social contact patterns, age and household structures, we also explored how epidemiological heterogeneities within the population may influence the spread of an infection. We incorporated different scenarios for susceptibility to infection when exposed and infectiousness when infected according to age. As anticipated given the social contact patterns and household structures, the susceptibility and infectiousness of children and adolescents were highly influential for the disease transmission in the whole population and in the parental generation in particular. Changes to these epidemiological parameters in the elderly population clearly affected that age group, but exerted much less influence on other age groups.

The elderly population, however, is affected differently by changes in the transmission parameters than the rest of the population, when subject to elevated susceptibility. As the probability of transmission given an effective contact increases, the underestimation of the attack rate in the elderly population when omitting age-dependent susceptibility (baseline scenario) increases, while it decreases in the rest of the population, and in all other scenarios. Generally, many older adults escape infection due to their limited number of contacts within and outside the household. However, as the transmission probabilities increase, a proportionately larger share of households with elderly people are reached by the infection and more individuals within the households become infected if their susceptibility is elevated. Thus, the impact of epidemiological heterogeneities (e.g. age-specific susceptibility) is not only dependent on the transmission parameters but also on other heterogeneities in the population, including social contact patterns and household structures.

Overall, we find that the strong relationship between age and household structures at the individual and population level, in combination with social mixing patterns and epidemiological parameters, shape the spread of an emerging infection. Disease transmission in the adult population in particular is influenced by differential household compositions. Moreover, we highlight how demographic processes alter population structures with differential impact on the disease transmission dynamics across population groups. Nevertheless, our study faces several limitations with regard to demographic modelling as well as infectious disease modelling.

We recognize that the demographic processes in the microsimulation are simplifications of highly complex processes and that the inherent uncertainty in population projections preferably is described in the form of probability distributions [43,44]. Moreover, the sensitivity in the association between demographic and epidemiological outcomes could have been explored. However, expanding our demographic microsimulation is not considered necessary to fulfil the aim of this paper, which is to document the impact of population structures and the changes therein on the spread of an emerging infection. For future research, however, it would be relevant to compare the microsimulation and two-level mixing model with other epidemic models with household-structured host population (e.g. [45]).

In our model of infectious disease transmission, we assume a fully susceptible population, restricting our study to emerging infections. Expanding the study to endemic infectious diseases requires not only information on age-specific patterns of prior immunity but also information on how immunity is distributed in households. Alternatively, a population at an endemic disease equilibrium can be generated, for example by simulating disease transmission over a long period of time before the actual analysis [11,13]. However, this would require a rather complex technique and/or detailed (historical) demographic data. Moreover, if the fertility and mortality schedules remain constant while generating an endemic disease equilibrium, the population eventually acquires the age distribution of the stable population associated with those underlying schedules of vital rates, which may not resemble the population of interest [25].

Another limitation in our model of disease transmission concerns the social contact patterns. If SPCs had been included in the social contact matrix, the incidence in the population of working age would have been slightly higher, however, the relationships found between population groups would remain. Additionally, we assume that the contact patterns in the community and within the households remain constant over time despite the demographic changes. Methods for adjusting social contact matrices to evolving demographic structures have been proposed, but these are not based on empirical evidence for how contact patterns behave over longer time frames as population structures evolve [46,47]. A comparison of two social contact surveys in Belgium five years apart suggests that the contact rates can be assumed stable, but the demographic change in this period is of course limited [2].

## Data Availability

The source code is available from the GitHub repository: https://github.com/signemoegelmose/demographic_microsimulation_EXTERNAL [48]. We do not have permission to share the demographic input data from the Belgian population registers. Supplementary material is available online [49].
